# Comparison of the ED50 of prophylactic butorphanol in preventing morphine-induced pruritus with or without palonosetron: a prospective, double-blinded, randomized dose–response trial using an up-down sequential allocation method

**DOI:** 10.1080/07853890.2024.2304671

**Published:** 2024-01-17

**Authors:** LiHong Sun, Lin Jin, Cuicui Jiao, LuYang Wang, Qi Xu, Hui Wu, XinZhong Chen

**Affiliations:** Department of Anesthesiology, Women’s Hospital, School of Medicine, Zhejiang University, Hangzhou, China

**Keywords:** Caesarean delivery, morphine, pruritus, butorphanol, palonosetron

## Abstract

**Background:**

Butorphanol has been used to reduce the incidence and severity of neuraxial morphine-induced pruritus. Palonosetron is a commonly used antiemetic for the prevention of postoperative nausea and vomiting. The aim of our study was to compare the effective dose in 50% of subjects (ED50) of intravenous butorphanol infusion with or without a single intravenous bolus of palonosetron for preventing pruritus induced by epidural administration of morphine.

**Methods:**

A total of 120 parturients were randomly assigned to receive an intravenous bolus injection of palonosetron plus continuous infusion of butorphanol (Group P + B) or an intravenous bolus of saline plus continuous infusion of butorphanol (Group B) after epidural administration of morphine. The antipruritic effect was graded as satisfactory (numerical rating scale (NRS) of pruritus ≤3) or unsatisfactory (NRS >3) within 48 h after morphine treatment. The first patient in each group received butorphanol infusion at a rate of 4 µg/kg/h. The infusion dose for each subsequent patient in the corresponding group was increased by 0.2 µg/kg/h after an unsatisfactory response or decreased by 0.2 µg/kg/h after a satisfactory response. The ED50 was calculated for each group and compared using up-down sequential analysis.

**Results:**

The ED50 (mean [95% confidence interval (CI)]) of the dose of intravenous butorphanol infusion for preventing moderate to severe pruritus was lower in Group P + B (3.29 µg/kg/min [3.25–3.34 µg/kg/min]) than in Group B (3.57 µg/kg/min [3.47–3.67 µg/kg/min]) (*p* < 0.05).

**Conclusions:**

Under the conditions of the present study, a prophylactic use of 0.25 mg palonosetron reduced the ED50 of prophylactic infusion of butorphanol by approximately 8% to achieve a satisfactory antipruritic effect after epidural morphine for post-caesarean analgesia.

## Introduction

Neuraxial administration of morphine, a hydrophilic μ-opioid receptor agonist with well-established effectiveness and a long duration of action, is one of the most effective methods for post-caesarean analgesia [1–3]. Pruritus, a common adverse effect of morphine, is difficult to treat. For patients who take neuraxial morphine for postoperative analgesia, preventive antipruritic drugs are often considered in clinical practice. Butorphanol is considered an appropriate prophylactic measure against epidural morphine-induced pruritus without damaging morphine’s analgesic efficacy in clinical use [4–6]. However, high dose of butorphanol can cause adverse effects such as dizziness, drowsiness. It would be valuable to find an adjunct drug to prevent pruritus and lower the dose of butorphanol, because by administering lower doses of butorphanol, the risk for side effects would presumably decreased. It has been proposed that serotonin 3 (5-HT3) receptor antagonists, which are widely used as first-line therapeutic agents for the prevention of postoperative nausea and vomiting, are potentially effective for treating pruritus [7–9]. Palonosetron, a potent and highly selective antagonist of 5-HT3 receptor, is commonly adopted for preventing postoperative nausea and vomiting [10]. The aim of the present study was to compare the effective dose in 50% of subjects (ED50) of butorphanol when given at a weight-based intravenous infusion rate for the prevention of epidural morphine-induced pruritus with or without a single intravenous bolus of palonosetron.

## Materials and methods

### Design and study subjects

This prospective, double-blinded, randomized dose–response clinical trial was approved by the Research Ethics Committee of Women’s Hospital, School of Medicine, Zhejiang University (Number: IRB-20210132-R). Written informed consent was obtained from all parturients. The trial was registered at the Chinese Clinical Trial Registry (ChiCTR) (www.chictr.org), and the registration number is ChiCTR2200055313. Written informed consent was obtained from all participants. Eligible patients were recruited to the study. Inclusion criteria included American Society of Anaesthesiologists physical status II, singleton pregnancy with term gestation (37–42 weeks), and scheduled caesarean section under combined spinal-epidural anaesthesia. Exclusion criteria included contraindications to regional anaesthesia; history of intolerance to or abuse of opioids; chronic pain syndrome or current opioid therapy; previous disease causing pruritus; body mass index >50 kg/m^2^; and critical factors, such as preeclampsia, eclampsia, and placenta previa.

### Study protocol

Based on a computer-generated random number sheet, patients were randomly allocated into Group B (intravenous administration of normal saline plus continuous intravenous pump of butorphanol) or Group P + B (intravenous administration of palonosetron plus continuous intravenous pump of butorphanol). An investigator (Hui Wu) who did not participate in any anaesthetic care or data collection opened the sealed envelopes, which contain the group assignments, and prepared the drug according to the group allocation.

All patients fasted for over 6 h and received no premedication. On arrival in the operating room, basic vital sings, including pulse oximetry, non-invasive blood pressure, and electrocardiogram were continuously monitored. After a brief settling period, baseline resting BP of the patient will be recorded by calculating the mean of three consecutive measurements with a difference of not more than 10%. Combined spinal-epidural anaesthesia procedure was performed in the left lateral position of patient. First, under local anaesthesia, epidural puncture was performed at the estimated L3-4 vertebral interspace with a 16-G needle, the arrival of the needle tip into the epidural space was identified by the method of ‘loss of resistance to air’. Second, spinal puncture was conducted with a 27-G pencil-tip needle. After ascertaining free flow of clear cerebrospinal fluid from the pencil-tip needle, 15 mg of hyperbaric ropivacaine diluted with 10% dextrose was slowly injected over 15 s. If there is no outflow of CSF from the spinal needle, it will be removed, the direction of the epidural needle will be adjusted and the spinal needle will be reinserted. If there is still no CSF outflow, both needles will be removed and the CSE procedure will be repeated. If the second attempt is also unsuccessful, the case will be withdrawn from the study and further clinical care will be at the discretion of the attending anaesthesiologist. At the same time of ropivacaine injection, 5 mL/kg of warmed lactated Ringer’s solution was infused for 20 min and then slowed down to maintain the patency of veins. Simultaneously, phenylephrine (0.5 µg/kg/min) was given intravenously to prevent hypotension. An epidural catheter was placed into the epidural space cephalally for 3–4 cm. The patients were then returned to the supine position with left uterine displacement. The height of sensory block was evaluated by assessing the loss of anterior midline needling sensation. Surgery was permitted to start when sensory block height reached higher than T6. Hypotension, defined as systolic blood pressure < 90 mmHg or a drop in systolic blood pressure by more than 20% from baseline, was treated with a 50 μg bolus of intravenous phenylephrine. Reactive hypertension, defined as an increase in systolic blood pressure by over 20% from baseline, was treated by immediately stopping the infusion of phenylephrine. Bradycardia (heart rate <50 beats/min) without hypotension was managed by stopping the infusion of phenylephrine, which was restarted when the heart rate increased back to over 50 beats/min. Bradycardia with hypotension was treated with an intravenous injection of 0.5 mg atropine. If nausea occurred during the operation, dexamethasone 5 mg was given intravenously.

At skin closure, 2 mg of morphine (diluted to a total volume of 2 mL), followed by a 1 mL normal saline flush, was injected *via* the epidural catheter, and then the epidural was removed. The patients were intravenously administered 10 mL of either 0.25 mg palonosetron (Group P + B) or normal saline (Group B), followed by a continuous intravenous pump of butorphanol for 48 h. The drug was administered by the attending anaesthesiologist who was blinded to the details of the solution. The initial dose of butorphanol was set as 4 µg/kg/h, the dose of butorphanol for next patient was determined by the response of the previous patient in the same group to butorphanol according to the up-down sequential allocation method. An effective response was defined as a numerical rating scale (NRS) pruritus score (a numerical score representing the intensity of pruritus on a scale from 0 to 10, with 0 for having no symptoms and 10 having worst imaginable symptoms [11] ≤ 3 points. If the response of the previous patient was effective, the dose of intravenous butorphanol for the next patient was decreased by 0.2 µg/kg/h. In contrast, the dose of intravenous butorphanol for the next patient was increased by 0.2 µg/kg/h. The maximum dose value was 5 µg/kg/h; if the next dose would have exceeded the maximum value, the dose no longer increased, and the former dose was maintained. Two researchers (Qi Xu and LuYang Wang), who were blinded to group assignments and the details of butorphanol dose, recorded the response of each patient and reported it to Hui Wu, who determined the dose and prepared drugs for the next case.

At each time point (6 h, 12 h, 24 h, 48 h) after epidural morphine injection, the severity and location of pruritus and pain were recorded. Postoperative pain was evaluated by NRS pain score (a 10 cm linear scale, with 0 and 10 labelled as ‘no pain’ and ‘worst pain imaginable’, respectively) [12, 13]. Severe pain (NRS pain score >6 points) was treated with 50 mg of intravenous tramadol. Moderate pain (3 points < NRS pain score ≤ 6 points) was treated with 500 mg of oral acetaminophen. Pruritus was treated with 100 mg of intravenous diphenhydramine at the patient’s request. Adverse effects, including sedation level, hypotension, bradycardia, postoperative nausea, vomiting, respiratory depression, dizziness, and urine retention, were also recorded. Sedation level was assessed using the Ramsay scale (1 = awake and alert, 2 = awake but drowsy, 3 = asleep but arousable, and 4 = not arousable) [14]. Respiratory depression was defined as SpO2 < 90% or respiratory rate <10 breaths/min. If respiratory depression occurred, patients were treated with face mask oxygen inhalation, the infusion of butorphanol was stopped, and 0.2 mg of naloxone was given intravenously. If the patient developed postoperative nausea and vomiting, 5 mg of dexamethasone was given intravenously. Urine retention was treated with indwelling catheterization. All adverse effects were treated at the request and with the consent of each parturient. At the end of surgery, patients were required to rate their satisfaction with the postoperative analgesia (1 = satisfied, 2 = moderate, and 3 = poor).

### Statistical analysis

The sample size of the current study was based on the method of up-down allocation [15]. Based on the results of previous literature and our pre-experiment, butorphanol can only decrease the severity of pruritus rather than completely prevent its occurrence. Besides, the starting dose may have been far from the ED50. Therefore, a larger sample size, which is 60 subjects per group, was determined in the present study. The ED50 value and 95% confidence interval (CI) for the butorphanol infusion rate were determined according to the method described by Dixon [16]. The number of effective and ineffective responses in each group was counted, and Probit regression analysis was used as backup and sensitivity analysis.

To estimate ED50 and its confidence interval and compare the ED50 value between the two groups, we used RStudio (version 4.2.1) with the package of ed50simulation (version:0.1.1) based on the method described by Dixon-Mood [15, 17]. Other statistical analysis was performed using IBM SPSS Statistics version 26. Continuous variables are summarized as the means ± SDs, and the Shapiro–Wilk test was used to test normality of distribution. Normally distributed variables were analyzed using Student’s t test. Categorical variables were aggregated into frequency counts and analyzed using χ2 test. Nonnormally distributed variables are expressed as medians (25th percentile, 75th percentile) and analyzed using Mann–Whitney *U* test. Two-tailed *P* values of < 0.05 were considered statistically significant.

## Results

**Figure 1.**
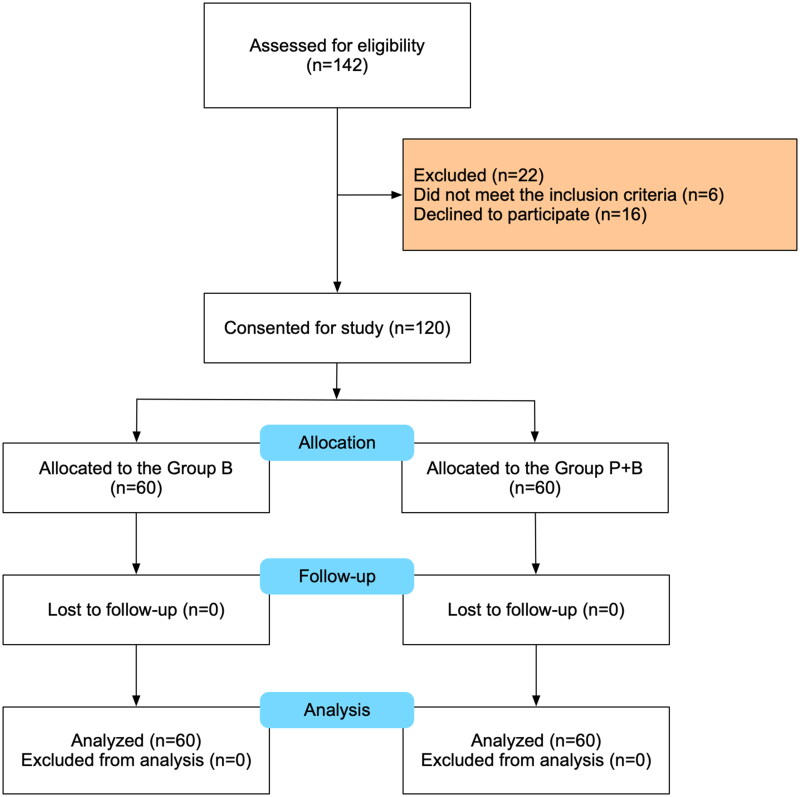
CONSORT diagram showing flow of patients in the study.

Eligibility was assessed for 142 patients scheduled for caesarean section between 11 January 2022 and 11 February 2023. In the process, 16 patients declined to participate, and 6 did not meet the inclusion criteria. 60 patients in each group were included in the final analysis. The Consolidated Standards of Reporting Trials (CONSORT) flow diagram is shown in Figure 1. The demographic and general characteristics were similar between study groups and are shown in Table 1.

**Table 1.** Demographic and obstetric characteristics.

	Group B (*n* = 60)	Group P + B (*n* = 60)	*p*-value
Age (years)	33.6 ± 4.3	32.2 ± 3.9	0.536
Height (cm)	159.4 ± 4.8	159.2 ± 4.7	0.788
Weight (kg)	66.4 ± 5.9	66.6 ± 6.7	0.834
Gestation (weeks)	38[38,39]	38[38,39]	0.909
Previous cesareans			0.836
0-1-2 (*n*)	43-17-0	45-15-0	

Data are presented as mean ± standard deviation or median [25th, 75th] for continuous variables or number for categorical variables.

**Figure 2.**
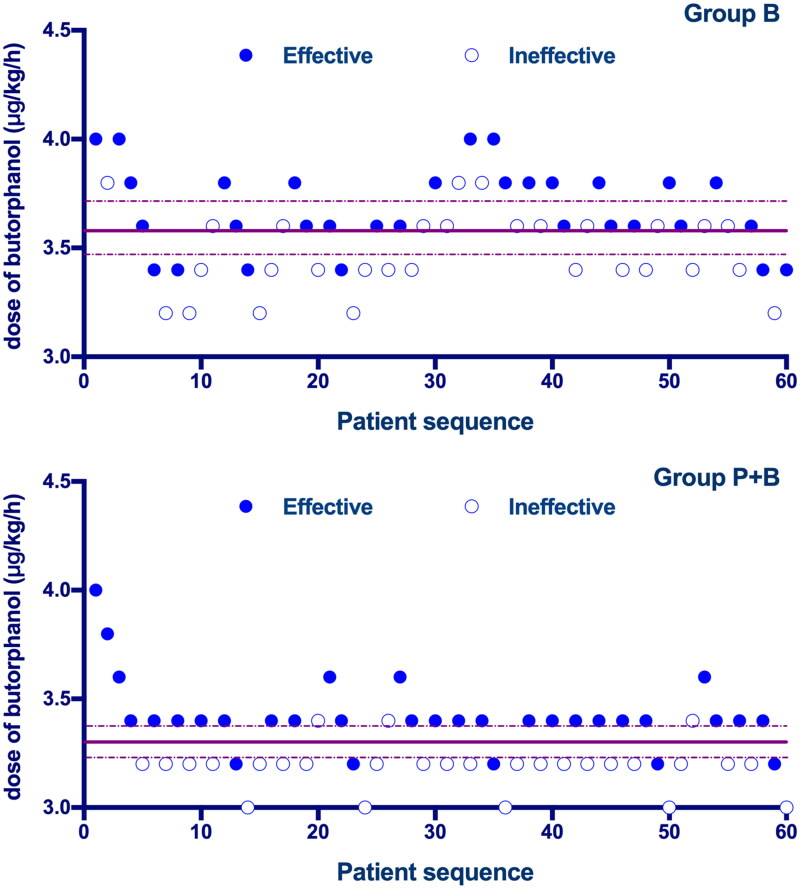
Individual response to prophylactic butorphanol at corresponding infusion rate (μg/kg/min). Solid lines represent the ED50 values, and dashed lines represent the 95% CI.

**Figure 3.**
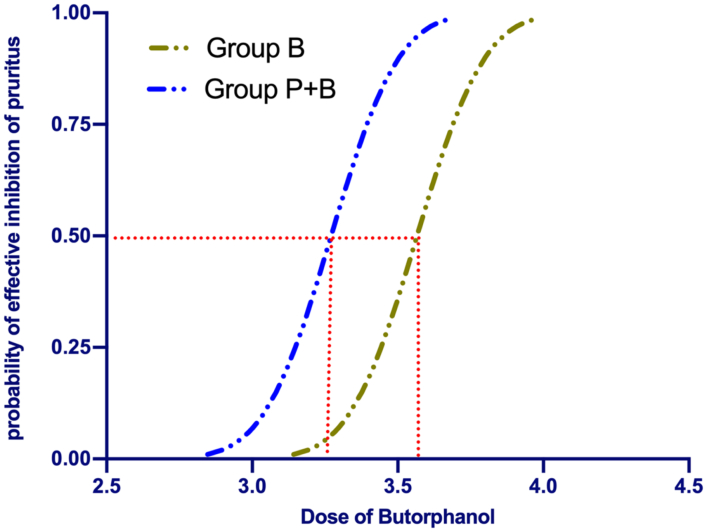
Dose–response curve of prophylactic butorphanol infusions for preventing pruritus plotted from estimated probabilities of effective response (1%–100%) versus the corresponding butorphanol infusion rate calculated using probit analysis.

The sequences of all the cases are presented in Figure 2. The ED50 value of the rate of intravenous butorphanol infusion for preventing moderate to severe pruritus was 3.57 µg/kg/min [3.47–3.67 µg/kg/min] in Group B and 3.29 µg/kg/min [3.25–3.34 µg/kg/min] in Group P + B. While using probit regression, the calculated ED50 of butorphanol infusion was 3.56 µg/kg/min [95% CI, 3.47–3.65 µg/kg/min] in Group B and 3.27 µg/kg/min [95% CI, 3.15–3.38 µg/kg/min] in Group P + B. There was a significant difference in the ED50 of butorphanol between the two groups (*p* < 0.05). The dose–response curves for butorphanol infusion in preventing moderate to severe pruritus are shown in Figure 3.

Characteristics of perioperative data in the two groups are presented in Table 2. There was no significant difference in the sensory block level; incidence of hypotension, bradycardia, nausea, and vomiting; surgery time; and estimated blood loss between the two groups. Neonatal characteristics, including Apgar scores and foetal weight, were comparable between the two groups.

**Table 2.** Perioperative data.

	Group B (*n* = 60)	Group P + B (*n* = 60)	*p*-value
Sensory block level T4/T5/T6] (n)	29/18/13	26/21/10	0.583
Hypotension (n)	10	12	0.637
Bradycardia (n)	2	3	>0.999
Nausea (n)	6	9	0.408
Vomiting (n)	0	1	>0.999
Neonatal weight (g)	3375 ± 366	3368 ± 425	0.915
Apgar score at 1 min	10 [10,10]	10 [10,10]	0.511
Apgar score at 5 min	10 [10,10]	10 [10,10]	>0.999
Surgery time (min)	50 [40.5, 64]	46 [38.25, 60]	0.111
Estimated blood loss (ml)	200 [200,300]	200 [200,300]	0.308

Data are presented as mean ± standard deviation or median [25th, 75th] for continuous variables or number for categorical variables.

The NRS pruritus score at 6 h in Group P + B was significantly lower than that in Group B (*p* = 0.022). Patients in Group P + B showed a lower incidence of pruritus in the head and face than those in Group B (*p* = 0.022). Patient satisfaction in Group P + B was higher than that in Group B (*p* = 0.034). There was no significant difference in pruritus score at 12, 24, and 48 h; pain score at 6, 12, 24, and 48 h; or requirement of assistant analgesia between groups (Table 3).

**Table 3.** Assessment of severity of pruritus and pain 6, 12, 24, and 48h after epidural morphine administration.

	Group B (*n* = 60)	Group P + B (*n* = 60)	*p*-value
Pruritus score at 6h	3 [0,4]	0 [0,4]	0.022
Pruritus score at 12h	2 [0,2]	0 [0,2]	0.38
Pruritus score at 24h	0 [0,0]	0 [0,0]	0.666
Pruritus score at 48h	0 [0,0]	0 [0,0]	0.242
Pruritus sites			
head and face	21	10	0.022
trunk	12	7	0.211
limbs	6	5	0.752
6h			
Pain at rest	0 [0,1]	1[0,1]	0.103
Pain with movement	2 [2,3]	2 [2,3]	0.338
12h			
Pain at rest	1 [0,1]	1[0,1]	0.222
Pain with movement	3 [2,3]	3 [2,3]	0.763
24h			
Pain at rest	0 [0,1]	0 [0,1]	0.326
Pain with movement	2 [1,2]	2 [2,2]	0.892
48h			
Pain at rest	0 [0,1]	0 [0,1]	0.783
Pain with movement	1 [1,2]	2 [1,2]	0.993
Assistant analgesia	10	12	0.637
Patient satisfaction 1-2-3 (I)	34-24-2	45-15-0	0.034

Data are presented as median [25th, 75th] for continuous variables or number for categorical variables.

Table 4 presents the incidence of adverse events after surgery. Compared with Group B, patients in Group P + B showed a significantly lower incidence of nausea (*p* = 0.002) and dizziness (*p* = 0.031). No cases of postoperative hypotension, bradycardia, vomiting, respiratory depression, and urinary retention occurred in both groups. (Table 4).

**Table 4.** Adverse events within 48h after surgery.

	Group B (*n* = 60)	Group P + B (*n* = 60)	*p*-value
Sedation level 1-2-3-4 (*n*)	54-6-0-0	55-5-0-0	0.752
Hypotension (*n*)	0	0	>0.999
Bradycardia (*n*)	0	0	>0.999
Nausea (*n*)	17	4	0.002
Vomiting (*n*)	0	0	>0.999
Respiratory depression (*n*)	0	0	>0.999
Dizziness (*n*)	15	6	0.031
Urinary retention (*n*)	0	0	>0.999

Data are presented as number for categorical variables.

## Discussion

This prospective, double-blinded, randomized dose–response trial is the first study focusing on the infusion dose of butorphanol for preventing morphine-induced pruritus in women having elective caesarean delivery. Our results showed that prophylactic administration of 0.25 mg palonosetron significantly decreases the required dose of butorphanol, reduces the severity of pruritus at 6h after epidural morphine administration, and lowers the incidence of post-operative nausea and dizziness. Butorphanol is considered an appropriate prophylactic measure against neuraxial morphine-induced pruritus. However, high doses of butorphanol can cause adverse reactions such as dizziness, drowsiness, nausea and vomiting, which might limit its clinical use [18]. Palonosetron, with few side effects and a high safety profile, is a commonly adopted medication to prevent or treat nausea and vomiting [19]. Since it is usually used in the perioperative period, it does not impose an additional or unnecessary financial burden on the patients. The results of the current study suggest that prophylactic use of palonosetron can reduce the dosage of butorphanol and its side effects, which has important clinical significance.

Although the effectiveness of epidural morphine for post-caesarean analgesia has been well established, morphine can produce undesired side effects, including pruritus, nausea, vomiting, urinary retention, and constipation [20, 21]. Pregnant women were reported to be more susceptible to neuraxial opioid-induced pruritus than other populations [22]. The amelioration of opioid-related side effects, especially pruritus, is one of the most important challenges in post-caesarean analgesia. It has been reported that naloxone, an MOR antagonist, can prevent neuraxial opioid-induced pruritus. However, the clinical application of naloxone as an antipruritic agent is limited due to its reversion and impairment of the analgesic effect of opioids [23]. In contrast, butorphanol, an opioid receptor agonist-antagonist, was demonstrated to be effective for pruritus prophylaxis without affecting the analgesic effects of morphine [24]. Butorphanol was recommended by the World Health Organization for adjunctive postoperative pain relief [25]. In addition, butorphanol has a very low concentration in breast milk and will not cause adverse effects on newborns [4, 26]. Currently, butorphanol is considered an appropriate prophylactic measure against epidural morphine-induced pruritus in clinical use. However, high dose of butorphanol can cause adverse effects such as dizziness, drowsiness, nausea, and vomiting [27]. Given that the serotonergic pathway is involved in the underlying mechanisms of opioid-induced pruritus [8, 22], the application of a 5-HT3 receptor antagonist might improve the efficacy of butorphanol in ameliorating pruritus. Therefore, the present study was initially conducted to determine the ED50 of butorphanol with and without palonosetron to prevent epidural morphine-induced pruritus.

Continuous infusion was chosen as the route of butorphanol administration due to its short duration of action. We chose palonosetron for its high affinity for the 5-HT3 receptor and longer plasma half-life (approximately 40h) than other antagonists, such as ondansetron (approximately 3.8 h) [28, 29]. The dose of palonosetron was set to 0.25 mg based on the combination of the literature results and our clinical observations. In a placebo-controlled trial, Chun et al. have found that intravenous palonosetron 0.075 mg could effectively reduce the incidence of postoperative nausea and vomiting (33% in the palonosetron group vs. 52% in the placebo group) during the 0-72 h period after surgery [30]. However, an incidence of 33% is still high for parturients who usually start breastfeeding shortly after surgery. Clinical trials have demonstrated that intravenous palonosetron at both 0.25 and 0.75 mg was generally well tolerated in preventing chemotherapy-induced nausea and vomiting with few adverse events [31]. Our study showed that 0.25 mg palonosetron was safe to use in parturients after caesarean surgery, with no reported adverse events related to palonosetron, which was consistent with the results of a previous study [32]. Sedation scores did not differ between the two groups, and no cases of respiration depression were observed in either group. These results demonstrated that 2 mg epidural morphine, 0.25 mg palonosetron, and all dose gradients of butorphanol adopted in our study were generally safe.

For the regimen and dose of neuraxial morphine, all the participants in this study received 2 mg epidural morphine. The dosage was set based on the protocol in similar studies and our routine clinical experience in practice. Epidural morphine is usually administered at a dose of 1 mg to 3 mg, which is approximately 10 times that in other studies using intrathecal morphine [21, 23]. For the assessment of pruritus, we recorded the intensity of pruritus at the timepoint of 6h, 12 h, 24 h, 48h after epidural morphine injection. The patients were asked to report the situation of pruritus during the time period between two evaluations. The effective dose was defined as pruritus score ≤ 3 at any time during the 48h period. In addition, we also collected data on specific body sites of pruritus. Typical sites of epidural morphine-induced pruritus mainly include the head and face, trunk, limbs, and occasionally even the whole body [33]. The lower incidence of pruritus in the head and face in Group P + B may be explained by the mechanism that neuraxial opioids induce pruritus by acting on 5-HT3 receptors in the spinal dorsal horn and trigeminal nucleus of the medulla [34].

Our study has some limitations. First, the dose of palonosetron used in the P + B group was fixed at 0.25 mg. Different doses of palonosetron might have different impacts on the ED50 of butorphanol. Second, our study compared the ED50 value for butorphanol infusions. However, in clinical practice, ED90 or ED95 is more preferable. Third, the study only consisted of parturients, the results may be different in other populations. To increase generalizability of the current findings, further studies are needed to focus on the dosage of palonosetron and butorphanol in preventing pruritus induced by epidural neuraxial administration of morphine in other patient populations.

## Conclusions

In summary, under the conditions of this study, prophylactic use of 0.25 mg palonosetron reduced the ED50 of butorphanol infusion by approximately 8% to achieve a satisfactory antipruritic effect after epidural administration of morphine in healthy parturients.

## Ethics approval and informed consent

This randomised, double-blinded study was approved by the Ethical Committee of Women’s Hospital, Zhejiang University School of Medicine (Hangzhou, China) (Approval No. IRB-20210132-R), and was registered prior to patient enrolment at the Chinese Clinical Trials (Registration No. ChiCTR2200055313, http://www.chictr.org.cn). Written informed consent was obtained from all participants. We confirm our study complies with the Declaration of Helsinki.

## Consent for publication

All authors have read and approved the manuscript, and agree to submit to the journal.

## Data Availability

The data supporting the study findings are available from the corresponding author upon reasonable request.
